# Evaluation of Sucrose Laurate as an Intestinal Permeation Enhancer for Macromolecules: Ex Vivo and In Vivo Studies

**DOI:** 10.3390/pharmaceutics11110565

**Published:** 2019-10-31

**Authors:** Fiona McCartney, Mónica Rosa, David J. Brayden

**Affiliations:** 1 UCD School of Veterinary Medicine and UCD Conway Institute, University, University College Dublin, Belfield, Dublin 4, Ireland; fiona.mccartney@ucd.ie; 2 Sublimity Therapeutics, DCU Alpha Innovation Campus, Dublin, Dublin 11, Ireland; monica.rosa@sublimitytherapeutics.com

**Keywords:** oral peptide delivery, oral insulin, intestinal permeation enhancers, sucrose laurate esters, formulation excipients

## Abstract

Oral delivery of macromolecules requires permeation enhancers (PEs) adaptable to formulation. Sucrose laurate (SL) (D1216), a food grade surfactant, was assessed in Caco-2 monolayers, isolated rat intestinal tissue mucosae, and rat intestinal instillations. Accordingly, 1 mM SL increased the apparent permeability coefficient (*P*_app_) of [^14^C]-mannitol and reduced transepithelial electrical resistance (TEER) across monolayers. It altered expression of the tight junction protein, ZO-1, increased plasma membrane potential, and decreased mitochondrial membrane potential in Caco-2 cells. The concentrations that increased flux were of the same order as those that induced cytotoxicity. In rat colonic tissue mucosae, the same patterns emerged in respect to the concentration-dependent increases in paracellular marker fluxes and TEER reductions with 5 mM being the key concentration. While the histology revealed some perturbation, ion transport capacity was retained. In rat jejunal and colonic instillations, 50 and 100 mM SL co-administered with insulin induced blood glucose reductions and achieved relative bioavailability values of 2.4% and 8.9%, respectively, on a par with the gold standard PE, sodium caprate (C_10_). The histology of the intestinal loops revealed little damage. In conclusion, SL is a candidate PE with high potential for emulsion-based systems. The primary action is plasma membrane perturbation, leading to tight junction openings and a predominant paracellular flux.

## 1. Introduction

Intestinal permeation enhancers (PEs) are present in the majority of solid-dose formulations currently in clinical trials for oral macromolecule delivery [1,2]. Permeation enhancers work to reversibly enhance epithelial permeability via the paracellular and/or transcellular route. First generation PEs include medium-chain fatty acids (MCFAs), MCFA derivatives, bile salts, acyl carnitines, and EDTA [3]. Though their mild perturbation action on intestinal epithelia in the high concentrations that are required in vivo is typical of surfactant-based detergents, the capacity of the intestine to undergo rapid epithelial cell turnover has not made the lack of specific mechanisms a barrier for the use of most of these PEs in oral peptide clinical trials to date [4]. Even so, it appears that oral relative bioavailability of peptides in humans is limited to ~1–2% with Generation 1 PEs [5], thus their application in once-a-day oral dosing must ideally be matched to stable macromolecules with long half-lives [6,7]. Although it would seem that tight junction-specific agents targeted at enzymatic pathways [8] or claudin-4 [9] might have less capacity for epithelial toxicity than agents that cause perturbation through non-specific actions, second-generation PE molecules for oral delivery remain in preclinical studies likely because of low stability, high cost as well as formulation and manufacturing issues. In addition, Pharma has an understandable aversion to the increased financial and regulatory risk associated with using new chemical entity PEs in oral macromolecule formulations—unless they can provide more than an incremental increase in efficacy.

**Figure 1:**
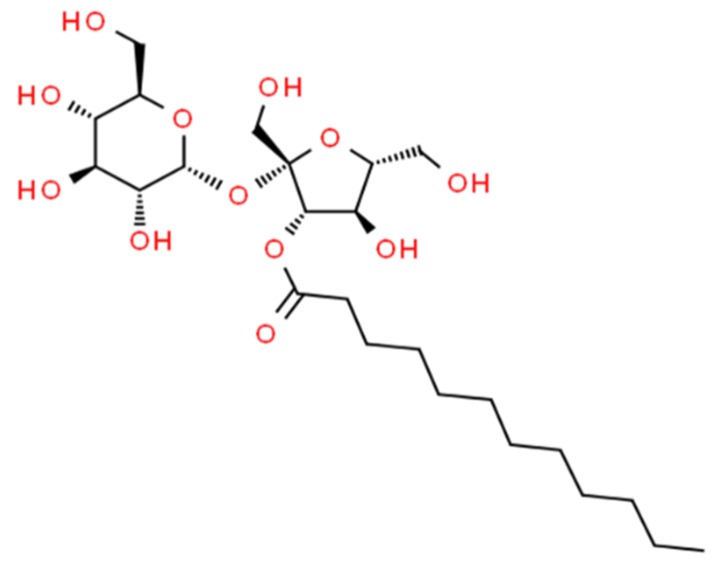
Structure of sucrose monolaurate (SL, Mw 525 Da). Sucrose laurate can also contain di/tri or polyesters [15].

With decades of preclinical and clinical research in Generation 1 MCFAs as intestinal PEs to call upon [10,11], our approach was to scan the FDA-approved food additives to see if MCFAs were already established in any formats suitable for oral formulation in lipid-based systems. Of the MCFAs, sodium laurate (C_12_) was the particular focus, because it was the most potent PE in a Caco-2 pre-screen using carbon chain lengths 6–14 [12]. Yet C_12_ does not feature in oral macromolecule formulations in clinical trials, because it has a low critical micellar concentration of 0.3 mM [13] and is quite cytotoxic in higher concentrations [12]. Moreover, due to the fact of its low solubility, it is difficult to formulate it in matrix tablets in comparison to the less potent gold standard PE in clinical trials—sodium caprate (C_10_). In the FDA food additive database, C_12_ is present in the format of a sucrose ester [14] (i.e., sucrose laurate (SL), Mw 525 Da (Figure 1)), which may offer better formulation possibilities compared to the salt form of the fatty acid.

Sucrose esters are non-ionic amphiphilic emulsifying surfactants with sucrose acting as the hydrophilic head and the fatty acid acting as the lipophilic tail. The hydrophilic–lipophilic balance (HLB) is defined by the MCFA carbon chain length, and C_12_ confers a value of 16 on SL, typical of oil-in-water emulsions. Sucrose esters are present in baking products, ice-cream, flavoured drinks, instant noodles, chewing gum, mayonnaise, as well as in the cosmetics industry [16]. The European Food Safety Authority (EFSA) adopted a very high acceptable daily intake of 40 mg/kg body weight per day in food for sucrose esters (E 473) [17], noting that current estimates of high exposure levels in humans are unreliable [18]. SL is made using green chemistry and is degraded to biodegradable metabolites by lipases and esterases [19]. It has potential for peptide formulation in promising lipid-based systems including oral self-emulsifying drug delivery systems (SEDDS) [20]; this is an attractive feature which delineates it from standard tablet formats and allows it to be considered for possible inclusion in oral formulations based on hydrophobic ion pairing [21].

The majority of research on SL has focussed on its capacity as a solubilising excipient [22]. To a lesser extent, a number of groups have examined it as a PE across several non-injectable routes. For example, it increased the permeability of ketoprofen and lidocaine across hairless mouse skin ex vivo [23] and increased flux of fluorescein isothiocyanate (FITC)-dextran 4000 (FD4) across cultured human nasal epithelial cell line monolayers [24]. Its action as a PE at the intestinal epithelium have been studied more widely in preclinical models. Kiss et al. [25] demonstrated that SL (D-1216) reduced transepithelial electrical resistance (TEER) in Caco-2 monolayers and enabled increased transepithelial flux of atenolol, fluorescein and P-glycoprotein (P-gp) substrates. They concluded that SL fluidised the plasma membrane and increased fluxes by both the paracellular and transcellular routes but without a direct effect on P-gp. However, the concentrations tested were low (up to 0.2 mM) Glynn et al. [26] confirmed a concentration-dependent TEER reduction across Caco-2 induced by SL (E-473) and noted the induction of lactate dehydrogenase release at similar concentrations, leading the authors to raise a concern over modification of tight junction integrity by food additives.

In examining the effects of SL with less reductionist approaches, Maher et al. [27] tested the capacity of the monoester version of SL along with other surfactants to increase the apparent permeability coefficient (*P*_app_) of [^14^C]-mannitol across isolated rat colonic mucosae mounted in Ussing chambers using a limited concentration range. They identified it as a good PE with efficacy on a par with bile salts in that model, but again they found it difficult to dissociate the flux increase from membrane damage at similar concentrations. The most relevant data on the PE effect of SL in the intestine have come from rat in situ instillation methods. Sucrose laurate (L-1695) increased absorption of alendronate across small intestinal and large intestinal closed loops in a concentration-dependent fashion, where 1% *w*/*v* was not damaging to the epithelium [28]. They also noted membrane fluidisation and hypothesised that a combination of increased paracellular and transcellular flux was likely. Yamamoto et al. [29] also used the in situ small intestinal loop method in rats to compare the permeation enhancement effect on absorption of FDs and 5(6)-carboxyfluorescein (CF) from a group of sucrose esters (i.e., SL, stearate, mystric, palmitate, and oleate) and found maximal efficacy with sucrose stearate and SL (L-1695), both at a concentration 0.5% *w*/*v*. Both studies saw good discrimination between concentrations that induced permeation enhancement and membrane damage. Others have confirmed the efficacy of SL (L-1695) on rat duodenal absorption of FD4 using instillations [30]. They found that SL (10% *w*/*v*) was as efficacious as C_10_ (10% *w*/*v*) and ascribed this to high water solubility. Apart from rectal administration, no intestinal instillation model has ever shown delivery of a peptide in the presence of SL to date.

In this study, our aim was to comprehensively explore the efficacy, cytotoxicity and mechanism of action of SL in three bioassays: Caco-2 monolayers (in vitro), isolated rat colonic tissue mucosae (ex vivo) and in rat jejunal and colonic instillations (in situ). For the most part, we used marker small molecules as permeants, but we report efficacy for the first time on delivery of insulin with SL in situ. High content analysis (HCA) of live cell imaging was also used to provide quantitative measures to sub-lethal exposures of SL to Caco-2 cells grown in 96 well plates and to provide further support for its mild detergent action consistent with excipient status.

## 2. Materials and Methods

### 2.1. Materials

Sucrose laurate (D1216) (PubChem CID: 5360776) was provided by Mitsubishi–Kagaku Foods Corporation (Tokyo, Japan). The D1216 was pharma grade, composed of 80% monoester with the remaining 20% made up of di-, tri- and polyesters. Sodium caprate (C_10_) (PubChem CID: 4457968) (Chemos, Regenstauf, Germany). Human insulin (Insuman^®^) was a gift from Sanofi (Frankfurt, Germany). The human insulin ELISA was obtained from Mercodia, Uppsala, Sweden. All cell culture and HCA reagents were obtained from Invitrogen™ Biosciences, Dublin, Ireland, unless otherwise stated. The Caco-2 was obtained from European Collection of Authenticated Cell Cultures (ECACC). General chemicals and reagents used were of analytical grade and were obtained from Sigma–Aldrich, Dublin, Ireland).

### 2.2. Apparent Permeability Coefficient (P_app_) of ^14^C-Mannitol across Caco-2 Monolayers Exposed to SL

^14^C-Mannitol is a small molecule probe for epithelial tight junction openings, and it was selected for initial Caco-2 studies in preference to FD4 due to the higher assay sensitivity. The Caco-2 cells from passages 52–60 were cultured in Dulbecco’s modified Eagle’s medium (DMEM) with 4500 mg/L glucose, 110 mg/L sodium pyruvate and sodium bicarbonate and phenol red (D6546), supplemented with 10% (*v*/*v*) heat-inactivated foetal bovine serum, 2 mM l-glutamine, 100 U/mL penicillin, 100 μg/mL streptomycin and 1% (*v*/*v*) non-essential amino acids. The viability of cells during sub-culture exceeded 90%, as determined by the exclusion of trypan blue with the Vi-CELL™ Series Cell Viability Analyzer (Beckman Coulter, Ireland). Cells were maintained in vented 75 cm^2^ flasks in a humidified cell culture incubator with 5% CO_2_ in air at 37 °C. The Caco-2 cells were seeded at a density of 3 × 10^5^ cells/well on Corning Costar tissue-culture-treated polyester Transwell^®^ filter inserts (pore size 0.4 μm, diameter 12 mm) and incubated at 37 °C with 5% CO_2_ in air for 21–28 days in DMEM [31]. The TEER was monitored throughout the culture period as a measure of monolayer differentiation and integrity using an EVOM™ voltammeter with a STX2 “chopstick” electrode (World Precision Instruments (WPI), Hertfordshire, UK). Acceptable monolayers from this source and passage range were required to have a minimum TEER of 1200 Ω·cm^2^, consistent with previous studies [32]. For [^14^C]-mannitol *P*_app_ determination, DMEM was replaced with 500 µL Hank’s Balanced Salt Solution (HBSS) supplemented with 4-(2-hydroxyethyl)-1-piperazineethanesulfonic acid (HEPES, 25 mM) and glucose (11 mM) on the apical side and 1500 µL HBSS on the basolateral side. The TEER was measured before and after HBSS replacement to ensure the monolayer integrity was not compromised. The TEER data are presented as the percentage TEER of the monolayer relative to the same monolayer at *T*_0_. ^14^C-mannitol, was added apically to all monolayers at a final concentration of 0.1 µCi/mL (56.5 mCi/mol). Fifty microlitres were sampled apically at *T*_0_ and replaced with either 50 µL of 10× SL stock or HBSS control solution. SL was added apically at concentrations of 0.05, 0.5 or 1 mM. The *P*_app_ of [^14^C]-mannitol across monolayers was measured by taking basolateral samples (750 µL) every 20 min up to 120 min. This time period is the minimum required to calculate a *P*_app_ and to obtain sufficient samples from the basolateral side; it is also the maximum period before monolayers start to deteriorate in HBSS. Samples were replaced with equal volumes of HBSS. At 120 min, HBSS was replaced with DMEM to allow for recovery in the incubator over 24 h, as monitored by TEER. [^14^C]-Mannitol containing samples were added to scintillation fluid (3 mL) and the disintegrations per minute (dpm) were measured using a liquid scintillation analyser (Packard Tricarb 2900 TR, Perkin Elmer, UK). The *P*_app_ (cm·s^−1^) was calculated according to the equation:

$$P_{app}=\frac{dQ}{dt}\frac{1}{A\cdot C_{0}}$$

where d*Q*/d*t* is the transport rate (dpm·s^−1^), A is the surface area of the cell monolayer (1.12 cm^2^), and *C*_0_ is the initial concentration in the donor compartment (dpm/mL) [31]. Each treatment was tested on three independent occasions with 2–3 replicates per plate.

### 2.3. MTS Assay of Caco-2 Cells Exposed to SL

An MTS (3-(4,5-dimethylthiazol-2-yl)-5-(3-carboxymethoxyphenyl)-2-(4-sulfophenyl)-2*H*-tetrazolium) assay was carried out on Caco-2 cells using the CellTiter 96^®^ AQueous One Solution Cell Proliferation Assay (Promega) [33]. The MTS is an end-point assay that detects living cells, but it does not reveal mechanisms. Caco-2 cells were seeded onto 96 well culture plates at a density of 2 × 10^4^ cells per well in DMEM and MEM and incubated for 24 h at 37 °C with 5% CO_2_ in air. Cells were incubated with SL (0.1–10 mM) or control buffer for 1 h and 24 h to assess acute and chronic exposure, respectively. Twenty microlitres of MTS reagent was added to each well and the plate was further incubated for 4 h. Absorbance was read at 490 nm (UVM 340 plate reader, ASYS Hitech Gmbh, Eugendorf, Austria). Percent viable cells exposed to SL were measured relative to the control medium and with Triton™ X-100 (Tx, 0.05% *w*/*v*) as a positive control. Experiments were repeated on three occasions using multiple comparisons on each plate.

### 2.4. Immunofluorescence of Tight Junction Proteins in Caco-2 Cells Exposed to SL

Monolayers of Caco-2 cells were incubated with SL, fixed with methanol and immunofluorescent staining was performed with a primary antibody raised against ZO-1 (Alexa Fluor^®^ 594 conjugate (ZO1-1A12)) (Thermo-Fisher, Dublin, Ireland). The Caco-2 cells were seeded at a density of 1.5 × 10^5^ cells per well in an 8 well Nunc™ Lab-Tek II chamber slides (Fisher, Dublin, Ireland) and incubated in 200 μL of DMEM for 21 days; media were changed every 2 days [34]. Treatments made up in Phosphate Buffered Saline (PBS) were added using 10× stock and exposure was for 2 h. Media were then removed, and cells were washed gently with ice cold PBS. Cells were fixed in methanol for 15 min and washed in PBS (×3). A 1% bovine serum albumin (BSA) solution was used to block the cells for 45 min. Cells were permeabilised by incubating with 0.1% Triton™ X-100 in PBS for 10 min. This was then removed, wells were washed in PBS and the antibody in 1% BSA was incubated in the dark for 2 h, followed by further washing. Slides were mounted using Dako fluorescence mounting media. Images were acquired (20× magnification) using a Zeiss Axioplan microscope (Oberkochen, Germany). Negative controls were run for each experiment, using 1% BSA instead of primary antibody.

### 2.5. High Content Analysis on Caco-2 Cells Exposed to SL

High content analysis (HCA) using live cell imaging was used to investigate the effects of SL on Caco-2 cellular parameters, particularly sub-lethal toxic effects [12]. Cells were seeded in 96 well plates at densities of 6 × 10^3^ cells per well for 1 h and 24 h exposures and incubated overnight at 37 °C with 5% CO_2_. Cells were seeded on 96 well plates avoiding the outer wells in order to reduce edge effects. Sucrose laurate was tested in the concentration range 0.05–10 mM. A dye mixture containing Hoechst 33342 (0.8 µM), Fluo-4 AM (1 µM), tetramethyl rhodamine methyl ester (TMRM, 20 nM) and TOTO-3 iodide 642/660 (1 µM) was used. Positive controls were added for each HCA parameter: 100 μM carbonylcyanide-*p*-trifluoromethoxyphenylhydrazone (FCCP) to decrease mitochondrial membrane potential (MMP), 20 μM ionomycin to increase cytosolic calcium (IC) and 0.05% (*v*/*v*) Triton™ X-100 to increase plasma membrane potential (PMP). Plates were imaged and analysed using an In-Cell^®^ 1000 High Content Analyzer (GE Healthcare, Buckinghamshire, UK).

### 2.6. [^14^C]-Mannitol and FD4 Fluxes across Isolated Rat Colonic Mucosae Exposed to SL

Ex vivo studies were carried out in accordance with University College Dublin Animal Research Ethics (AREC) protocol #14-28, approved 7 January 2014. Adult male Wistar-CRL rats were obtained from the Biomedical Facility, UCD and the Charles River Laboratory, UK, and were housed in a pathogen-free environment with controlled conditions of humidity and temperature under a 12:12 h light/dark cycle with access to laboratory chow and filtered water ad libitum. Ussing chamber studies were carried out as previously described [35]. Rats weighing 250–400 g were euthanized by stunning and cervical dislocation. Midline laparotomy was performed and the colon was excised and placed directly in Krebs-Henseleit (KH) buffer. With the colon facing basolateral side up, the muscle layer was removed with blunted #5 tweezers, and the tissue was mounted in the pre-equilibrated Ussing chambers with a circular diameter of 0.63 cm^2^ (WPI, Herfordshire, UK) [36]. The transepithelial potential difference (PD, mv) and short-circuit current (*I*_sc_, μA) were determined and TEER was calculated using Ohm’s law. [^14^C]-Mannitol (0.1 µCi/mL) and FD4 (2.5 mg/mL) were added apically and samples collected every 20 min for 2 h. Samples containing [^14^C]-mannitol were analysed for radioactivity as described in Section 2.2. The FITC signal intensity was measured using a Spectra Max Gemini fluorescence intensity microplate reader (Molecular Devices, San Jose, CA, USA) at excitation/emission wavelengths of 490 nm/525 nm. Sucrose laurate (1.5–10 mM) was added to the apical side of tissue mucosae; sodium caprate (C_10_, 10mM) was used as a positive control. After 2 h exposures, *I*_sc_ responses to carbachol (CCh, 0.1–10 µM) were used to assess functional epithelial electrogenic ion transport [35]. The *P*_app_ values were calculated as described in Section 2.3. Muscle-stripped jejunal mucosae are not assessed in flux studies because the tissue is too delicate to be used for comparative analyses with PEs that cause membrane perturbation [35].

### 2.7. Rat Jejunal and Colonic In Situ Instillations

Procedures were performed under licence AE18982/P036 from the Irish Health Products Regulatory Authority (HPRA) approved 6 October 2014. Rat specifications and housing was as described Section 2.6. All procedures were carried out under anaesthesia induced with isoflurane gas (Iso-Vet, 1000 mg/g isoflurane liquid for inhalation (Piramal Critical Care, Middlesex, UK)) at the rate of 5 L/min mixed with 4 L/min O_2_ in an induction box and then maintained at 2–2.5 L/min mixed with 1 L/min O_2_ via a mask using an anaesthesia vaporising unit (Blease Medical Equipment Ltd., Chesham, UK). In situ instillations were performed as previously described [36], but with minor modifications. Briefly, following a midline laparotomy, the jejunum or colon was tied off at both ends 5–7 cm apart with a size 4 braided silk suture to create a loop. Insulin solutions (50 IU/kg) were injected into the lumen with and without SL (50 or 100 mM) using a 1 mL syringe with 30 G needle. In some studies, C_10_ (50 and 100 mM) was used as a gold standard positive control PE. Glucose levels were determined using a glucometer (Accu-chek Aviva, Roche). Retro-orbital blood samples were taken at T0, 20, 40, 60, 80, 100, and 120 into 0.5 mL Eppendorf tubes and stored at 2–8 °C prior to centrifugation (6500× *g*, 5 min) and serum collection. Serum was stored at −20 °C until analysis for insulin using the Human Insulin ELISA kit (Mercodia, Uppsala Sweden). Animals were euthanized at the end of the experiment with intra-cardiac injection of 0.4 mL pentobarbital sodium (EUTHATAL™, Merial Animal Health Ltd., Woking, UK). To measure relative bioavailability (*F*, %) of insulin, one group was dosed with 1 U/kg insulin by s.c. injection. The following equation was used to calculate relative *F*:

$$Relative\;\%\;F=\frac{{AUC}_{\left(\mathrm{inst}.\right)}\times {Dose}_{\left(s.c.\right)}\;}{{AUC}_{\left(s.c.\right)}\times {Dose}_{\left(\mathrm{inst}.\right)}\;}\times 100$$

where *AUC*_(inst.)_ is the area under the serum concentration curve over the 120 min instillation period and *AUC*_(s.c.)_ is the area under the serum concentration versus time (0–120 min) after s.c. administration.

Pharmacological availability (%PA) of insulin was calculated using the equation:

$$Relative\;\%\;PA=\frac{{AAC}_{\left(\mathrm{inst}.\right)}\times {Dose}_{\left(s.c.\right)}\;}{{AAC}_{\left(s.c.\right)}\times {Dose}_{\left(\mathrm{inst}.\right)}\;}\times 100$$

where *AAC*_(inst.)_ is the area above the blood glucose curve over the 120 min instillation period and *AAC*_(s.c.)_ is the area above the blood glucose versus time (0–120) after s.c. administration of 1 IU/kg.

### 2.8. Histology of Rat Intestinal Tissue Mucosae Exposed to SL

Colonic epithelial sheets from Ussing chamber studies and jejunal and colonic tissue from in situ instillations were assessed. Tissues were fixed in 10% *w*/*v* formalin and embedded in paraffin wax. Five-micromillimeter tissue sections were cut on a microtome (Leitz 1512; GMI, USA) mounted on adhesive coated slides and stained with haematoxylin/eosin (H & E) and Alcian blue. Sections were examined by light microscopy (Nikon Labphoto; Kingston upon Thames, UK).

### 2.9. Statistical Analysis

Statistical analysis was carried out using Prism-5^®^ software (GraphPad^®^, San Diego, CA, USA). Analysis was carried out using two-way ANOVA with Bonferroni’s post-hoc test for electrophysiological measurements and for insulin data in rat studies and by one-way ANOVA with Dunnett’s post-hoc test for *P*_app_, MTS and HCA comparisons. The results are presented as the mean ± standard error of the mean (SEM). A significant difference was considered present if *p* < 0.05.

## 3. Results

### 3.1. Effects of SL on TEER and Permeability across Caco-2 Monolayers

**Figure 2:**
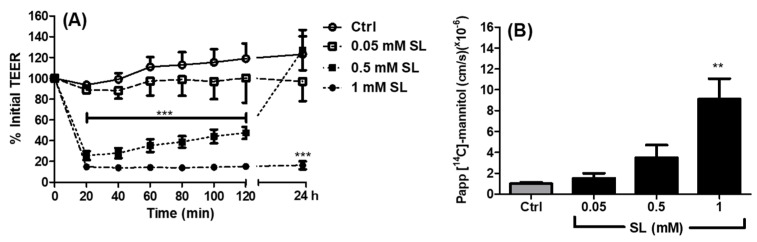
Effect of SL on (**A**) Transepithelial electrical resistance (TEER) across Caco-2 monolayers. Bar denotes significance from 20–120 min for 0.5 mM and 1 mM SL at the *p* < 0.001 level compared to the Hank’s Balanced Salt Solution (HBSS) controls (***). (**B**) the apparent permeability coefficient (*P*_app_) of ^14^C-mannitol across the Caco-2 monolayers; ** *p* < 0.01 compared to the HBSS controls. *n* = 3 per group.

Permeation-inducing effects of SL were confirmed using Caco-2 monolayers on Transwells^®^. The basal TEER of monolayers was 2000 ± 15 Ω·cm^2^, within the published range by this lab and others [37,38]. Monolayers were exposed apically to 0.05, 0.5, and 1 mM SL for 120 min before the treatments were removed and then monolayers were re-incubated in fresh culture media. Neither control monolayers exposed to medium alone nor monolayers exposed to 0.05 mM SL displayed reduction in TEER. However, 0.5 mM SL reduced TEER to a nadir at 20 min, which was fully reversed after 24 h recovery in DMEM (Figure 2A). 1 mM SL also reduced TEER for 20 min, but it was not reversible. The basal *P*_app_ for ^14^C-mannitol in control monolayers was 0.99 ± 0.10 × 10^−6^ cm·s^−1^ in the apical-to-basolateral direction. The *P*_app_ increased in the presence of 1 mM SL to 9.10 ± 2.0 × 10^−6^ cm·s^−1^, a 9-fold increase (Figure 2B). A 3.5-fold increase was also seen with 0.5 mM SL, but this was not statistically different and perhaps less efficacious because the TEER had already begun to reverse its reduction from 20 min onwards at this concentration. Overall, the effects of SL on TEER and the *P*_app_ were concentration-dependent and indirectly suggested that the paracellular pathway was affected by SL.

### 3.2. Effect of SL on ZO-1 Immunofluorescence in Caco-2 Cells

**Figure 3:**
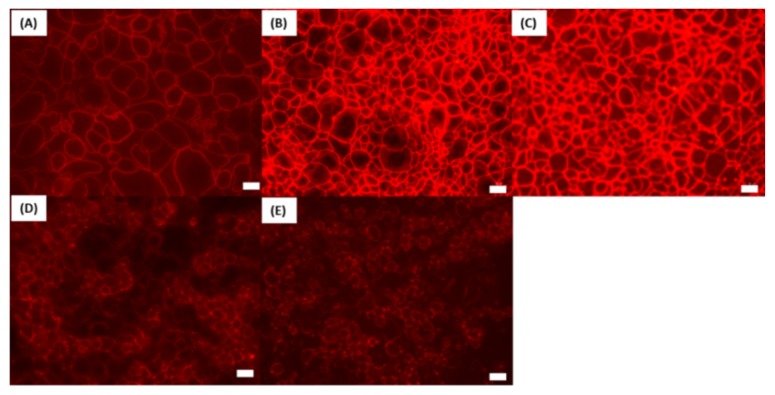
Representative immuno-fluorescence analysis of ZO-1 exposed to sucrose laurate (SL) compared to the Phosphate Buffered Saline (PBS) control. (**A**) Control, (**B**) 0.05 mM, (**C**) 0.1 mM, (**D**) 0.5 mM, (**E**) 1 mM SL. Bar = 10 µm.

In order to investigate the effects of SL ester on tight junction proteins, immunofluorescence was used. The Caco-2 cells were probed with an antibody ZO-1 (Figure 3). In the controls exposed to HBSS, ZO-1 presented in a continuous manner at the borders between cells. With 0.5 and 1 mM SL, this was not continuous and, in some areas, disruption in the immunostaining for ZO-1 was observed. Since SL increased monolayer permeability, it might enable the antibody to better access ZO-1, thus this result should be treated with caution. Overall, these results suggest that SL affects this tight junction protein at concentrations of 0.5 mM and 1 mM. At these concentrations, however, some histological damage to the cells was observed, so it was not possible to discriminate a discrete action on tight junctions from perturbation using antibody detection.

### 3.3. MTS and High Content Analysis (HCA) Studies in Caco-2 Cells

**Figure 4:**
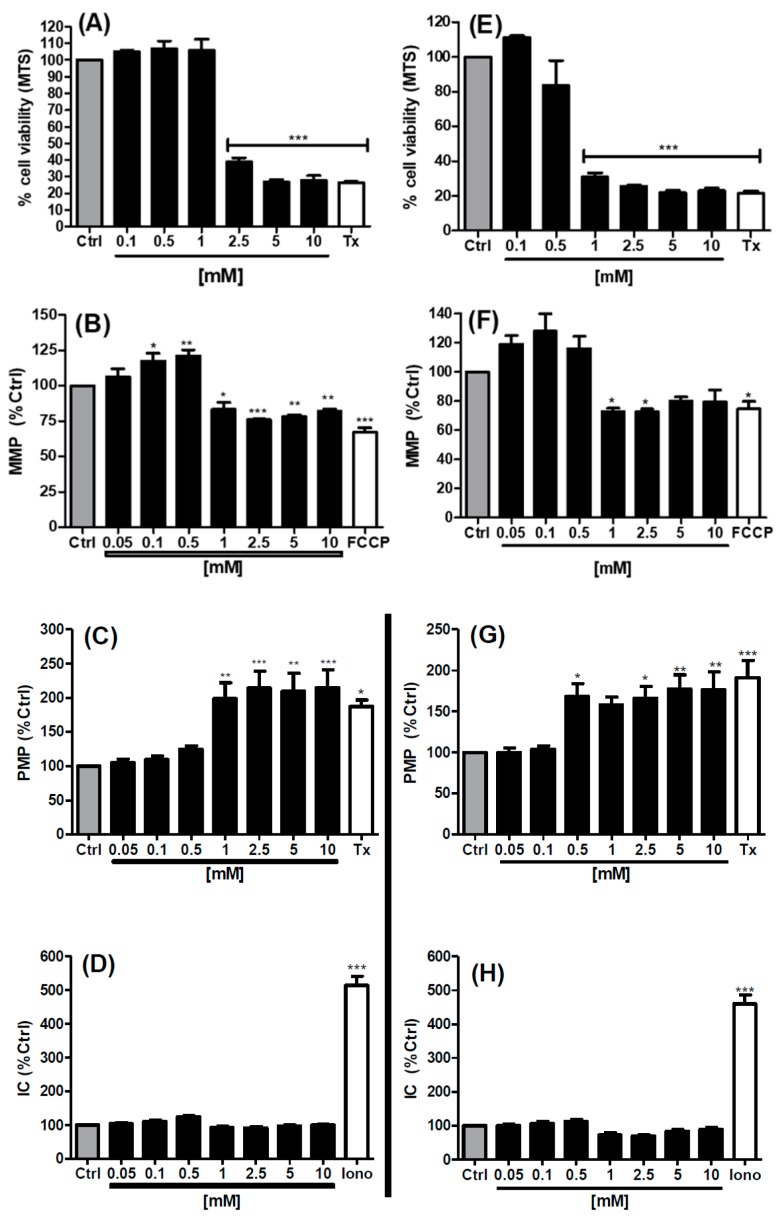
Effect of sucrose laurate (SL) on cell viability (MTS assay) and HCA parameters in Caco-2 cells at two exposure time points. The SL concentration range is 0.05–10 mM in each panel. (**A**) MTS (60 min), (**B**) MMP (60 min), (**C**) PMP (60 min), (**D**) IC (60 min), (**E**) MTS (24 h), (**F**) MMP (24 h), (**G**) PMP (24 h), (**H**) IC (24 h). Positive controls: FCCP (100 μM) for MMP, Tx (0.05% *w*/*v*) for MTS and PMP, and ionomycin (Iono, 20 μM) for IC. * *p* < 0.05, ** *p* < 0.01, *** *p* < 0.001, compared to the medium control (Ctrl). *n* = 3 per group.

The [^14^C]-mannitol flux studies suggested that the 1 mM concentration of SL may be somewhat cytotoxic because TEER reductions were not recoverable. The Caco-2 cell viability was assessed using the MTS cytotoxicity assay following 1 h and 24 h exposures to SL across a concentration range of 0.1–10 mM. 1 mM did not alter cell viability at 1 h, but it reduced it to 31% of the control value at 24 h exposure. At 2.5 mM SL, viability was reduced to 39% at 1 h and 26% at 24 h (Figure 4A,E). These data indicated that the 1 mM SL concentration which increased *P*_app_ across the monolayers over 2 h was not cytotoxic to Caco-2 cells but that even a slight increase in the concentration above 1 mM and an exposure time beyond 2 h would be. This reflects the limitations of testing PEs in Caco-2, where it is difficult to discriminate a true PE effect from cytotoxicity.

High content analysis (HCA) was used to further investigate the sub-lethal effects of SL across the concentration range of 0.05–10 mM on Caco-2 cellular parameters at 1 h and 24 h exposures (Figure 4B–D (60 min) and 4F–H (24 h). Mitochondrial membrane potential (MMP) and plasma membrane potential (PMP) parameter differences from medium controls were seen with 1 mM SL at 1 h and 24 h. The patterns of the changes for MMP exposed to SL showed a trend of increases at low concentrations (0.1–0.5 mM) followed by reductions at concentrations of 1 mM and above (Figure 4B,F). For PMP, the changes were restricted to increases at concentrations at a threshold of 1 mM at 1 h and at a threshold of 0.5 mM at 24 h (Figure 4C,G). The increases in PMP in cells seen at 1 mM SL were consistent with the increases in *P*_app_ in monolayers at this concentration. Importantly, SL had no effect on intra-cellular calcium (IC) at the concentrations and exposure times tested (Figure 4D,H). Focussing on the 1 mM concentration required for PE action in monolayers, which was not overtly cytotoxic, the HCA revealed that this concentration alters the potential difference of both the plasma membrane and mitochondrial membrane over a similar exposure timeframe, which may denote pre-apoptotic events.

### 3.4. Effects of SL on Rat Colonic Tissue Mucosae Mounted in Ussing Chambers

**Figure 5:**
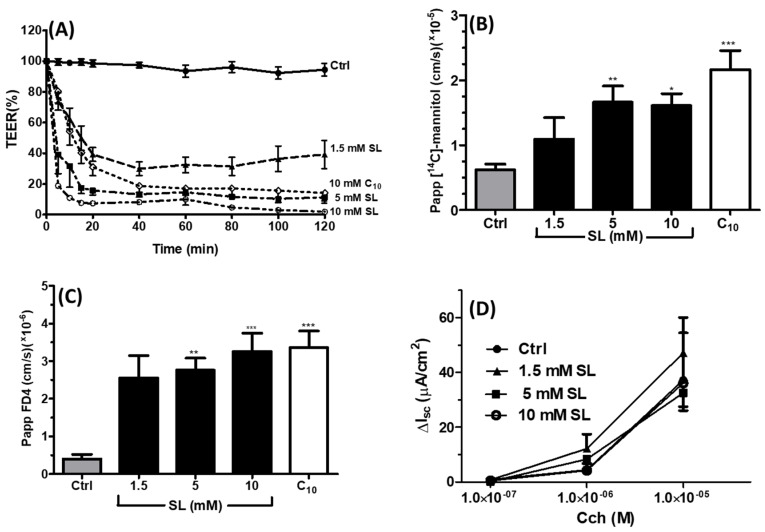
Effect of sucrose laurate (SL) on parameters of colonic mucosae ex vivo. (**A**) Transepithelial electrical resistance (TEER) (see Table S1 for statistical analyses), (**B**) *P*_app_ of [^14^C]-mannitol, (**C**) *P*_app_ of (FITC)-dextran 4000 (FD4), (**D**) *I*_sc_ changes in response to carbachol (Cch). Each value represents the mean ± SEM. Control group: *n* = 11; treatment groups: *n* = 3–7. Concentrations of SL are given on the *x*-axis; C_10_ (10 mM) was included as a positive control in (**A**–**C**). * *p* < 0.05, ** *p* < 0.01 and *** *p* < 0.001, compared to the control.

The TEER and *P*_app_ coefficients were measured across rat colonic mucosae after apical-side incubation with 1.5 mM, 5 mM, and 10 mM SL over 120 min. Colonic tissue was used in preference to small intestinal tissue mucosae due to the ease of reproducible epithelial dissection and more robust viability in chambers. With 1 mM SL emerging as the threshold for increasing flux in Caco-2 monolayers, we expected that higher concentrations would be needed to be efficacious in this bioassay and, thus, we tested an additional flux marker (FD4) as well as [^14^C]-mannitol. The C_10_ was also used as the gold standard PE in order to provide comparative data. The mean basal TEER in Krebs-Henseleit (KH) buffer for colonic tissue was 111 ± 6 Ω·cm^2^, above the cut-off value of 70 Ω·cm^2^ and similar to those obtained in published studies [39,40]. Apical addition of SL caused a concentration-dependent rapid decrease in TEER (Figure 5A). For concentrations of 1.5–10 mM SL, reduction in TEER was seen within 5 min. 1.5 mM and 5 mM SL caused similar TEER reductions as C_10_ (10 mM) in terms of rate and efficacy. Statistics for Figure 5A are given in Table S1. The basal *P*_app_ values for mucosae incubated in KH buffer were 0.62 × 10^−5^ cm·s^−1^ and 0.40 × 10^−6^ cm·s^−1^ for [^14^C]-mannitol and FD4, respectively, in the apical-to-basolateral direction. Sucrose laurate caused a concentration-dependent increase in the *P*_app_ of both flux markers, which was significant at 5 mM (Figure 5B,C). 10 mM of SL caused a 2.6-fold increase in the *P*_app_ of [^14^C]-mannitol compared to a 3.5 fold increase for C_10_. For FD4, 10 mM SL caused a similar fold increase compared to that induced by 10 mM C_10_—8.2 versus 8.4. In the presence of SL, colonic tissue responded to the basolateral addition of carbachol with an increase in *I*_sc_, indicative of electrogenic chloride secretion [41]. Large increases in *I*_sc_ in response to carbachol were still present in the presence of the three concentrations of SL (Figure 4D), confirming that the mucosae retained ion transport functional capacity. C_10_ was not included in the *I*_sc_ study, because it was previously shown to have a confounding interaction with carbachol [35].

**Figure 6:**
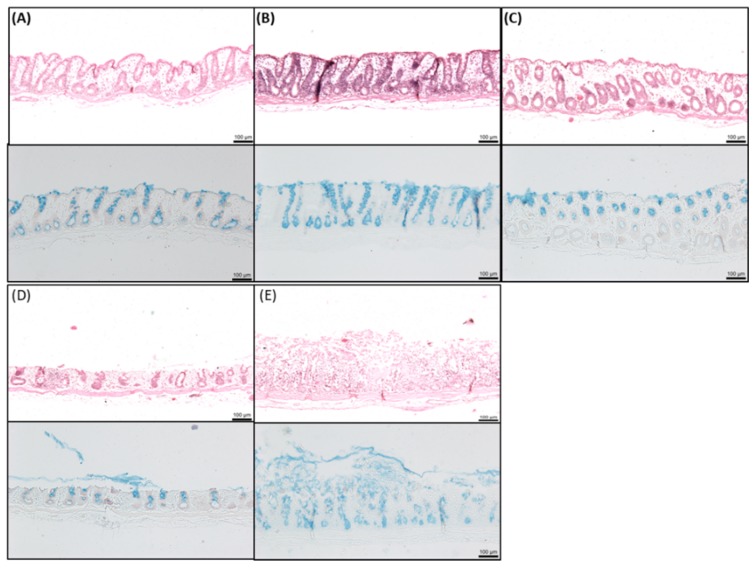
Representative histology of colonic tissue mucosae after 120 min exposure to sucrose laurate (SL) (1.5 mM, 5 mM, and 10 mM). H & E staining (upper panels, (**A**–**E**)) and neutral red and alcian blue staining (lower panels). Bar = 100 μm. (**A**) KH control; (**B**) 1.5 mM SL; (**C**) 5 mM SL; (**D**) 10 mM SL; (**E**) 10 mM C_10_.

The histology of colonic mucosae was examined following exposure to the three concentrations of SL over 120 min in chambers (Figure 6). Controls exposed to KH buffer were undamaged and a healthy intact epithelium was observed (Figure 6A). 1.5 mM SL caused no changes to the histology and was similar to the control (Figure 6B). Mucosae exposed to 5 mM SL displayed minor oedema, and some cell sloughing can be detected at the top of the cells (Figure 6C). With 10 mM SL, the level of damage at the top of the cells was further increased and a layer of mucus was detected on the apical side (Figure 6D). By comparison, mucosae exposed to C_10_ displayed clear evidence of significant perturbation and damage (Figure 6E). Overall, despite the evidence of some perturbation at high concentrations of SL, mucosae appeared intact, and this was consistent with the retention of electrogenic ion transport function.

### 3.5. Effects of SL on the Pharmacodynamics (PD) and Pharmacokinetics (PK) of Co-Administered Insulin in Jejunal and Colonic Instillations

**Figure 7:**
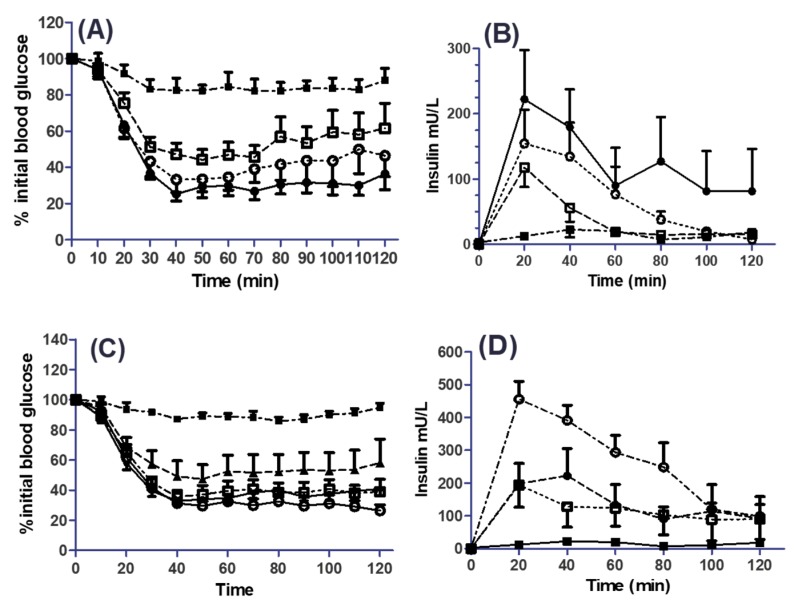
Intra-jejunal (i.j.) and intra-colonic (i.c.) instillations of Sucrose laurate (SL) with insulin (50 IU/kg). (**A**) Blood glucose (i.j.). Symbols: **☐** insulin/SL (50 mM); ○ insulin/SL (100 mM); ● insulin/C_10_ (100 mM); ■ insulin. (**B**) Plasma insulin (i.j.). Symbols same as in (**A**). (**C**) Blood glucose (i.c.). Symbols: ▲ insulin/SL (1 mM); ≤insulin/SL (10 mM); ○ insulin/SL (25 mM); ● insulin/C_10_ (25 mM); ■ insulin. (**D**) Serum insulin (i.c.). Symbols same as in (**C**) but without SL (1 mM). Data are shown as mean ± SEM of *n* = 6 in each group for each panel. For statistical analysis of each panel, see Table S2 (Figure 7A), Table S3 (Figure 7B), Table S4 (Figure 7C) and Table S5 (Figure 7D).

In situ rat intestinal instillations were carried out in the presence and absence of SL. The PBS and insulin solution (50 IU/kg) were used as controls with C_10_ used as a comparator in both regions. By the s.c. route, 1 IU/kg insulin caused a mean reduction in blood glucose from the value at *T*_0_ to 31% at *T*_90_ min, while PBS had no effect on blood glucose (Figure S1A). Serum insulin levels from s.c.-administered insulin increased to a *C*_max_ of 111 mU/L and a *T*_max_ of 20 min (Figure S1B, Table 1). Intra-jejunal instillation of insulin in PBS solution had no effect on serum glucose concentrations (Figure 7A). The SL and C_10_ were instilled intra-jejunally with insulin. Both agents caused a reduction in blood glucose (Figure 7A). Insulin with 100 mM SL caused a greater reduction in blood glucose from 20 min versus 30 min compared to 50 mM SL. Statistics from Figure 7A are given in Table S2. From 40 min onwards, 100 mM SL and 50 and 100 mM C_10_ reduced blood glucose in a similar manner and induced similar % PA values (2.4, 2.8 and 2.8), respectively (Table 1). Figure 7B shows the effect of SL on serum insulin levels. Insulin administered with either 50 or 100 mM SL had a shorter *T*_max_ compared to C_10_ (20 min and 30 min, respectively, compared to 43 min). 50 mM C_10_ induced the highest *C*_max_ of 276 mU /L, with an *AUC*_(0–120)_ of 15,210 mU/L·min (Table 1 and Figure S2).

With regard to intra-colonic instillations, enhancement effects of SL and C_10_ were greater for intra-jejunal instillations for both agents and at lower concentrations. Insulin administered with SL significantly decreased blood glucose at all concentrations till *T*_120_. 1 mM SL reduced blood glucose from 30 min to 120 min, with a % PA of 1.9 (Figure 7C, Table 1). It also increased serum insulin with the longest time to *T*_max_ (73 min), lowest *AUC*_(0–120)_ (9382 mU/L.min), lowest *C*_max_ (179 mU/L) and produced a relative % *F* of 2.7 (Table 1). Maximal glucose reductions were seen with lower concentrations of SL in the colon compared to the jejunum. 25 mM SL performed the best of all SL concentrations and out-performed C_10_ (25 mM) by the colonic route. For completeness, data are also shown for the admixtures of insulin with C_10_ (10 mM and 50 mM) (Figure S2). 25 mM SL induced a *C*_max_ of 458 mU/L, *T*_max_ of 23 min, *AUC*_(0–120)_ of 30,954 mU/L·min, and a relative % *F* of 8.9 (Figure 7D, Table 1). 50 mM SL induced a *T*_max_ of 33 min and lower values for all other parameters. It is interesting to note that 25 mM and 50 mM SL induced similar % PA values for insulin when delivered intra-colonically, 2.7% and 2.9%, respectively (Table 1). This suggests that 25 mM may be the optimum SL concentration for colonic delivery of insulin. The PK data confirmed that the permeation enhancing effects of SL on insulin absorption was substantially increased in the colon compared to jejunum (Figure 7B,D; Table 1). Overall, the PK and PD data indicate that SL performs on a par with C_10_ in both regions.

**Figure 8:**
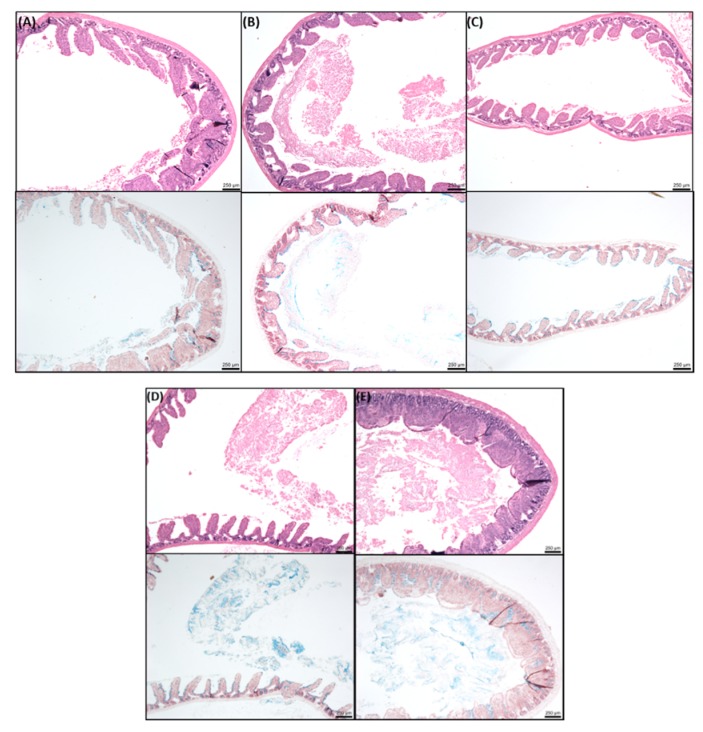
Representative histology obtained after intra-jejunal instillations for 120 min. H & E (upper panels, (**A**–**E**)) and alcian blue (lower panels). (**A**) insulin (50 IU/kg); (**B**) insulin/50 mM SL; (**C**) insulin/100 mM SL; (**D**) insulin/C_10_ (50 mM); insulin/C_10_ (100 mM). Bar = 250 µm.

Figure 8 shows the histological effects of SL in jejunal tissue following instillations over 120 min. The SL ad-mixed insulin induced morphological changes that were similar to insulin controls. Minor cell sloughing was observed, and increased levels of mucus were seen in the treated tissue.

**Figure 9:**
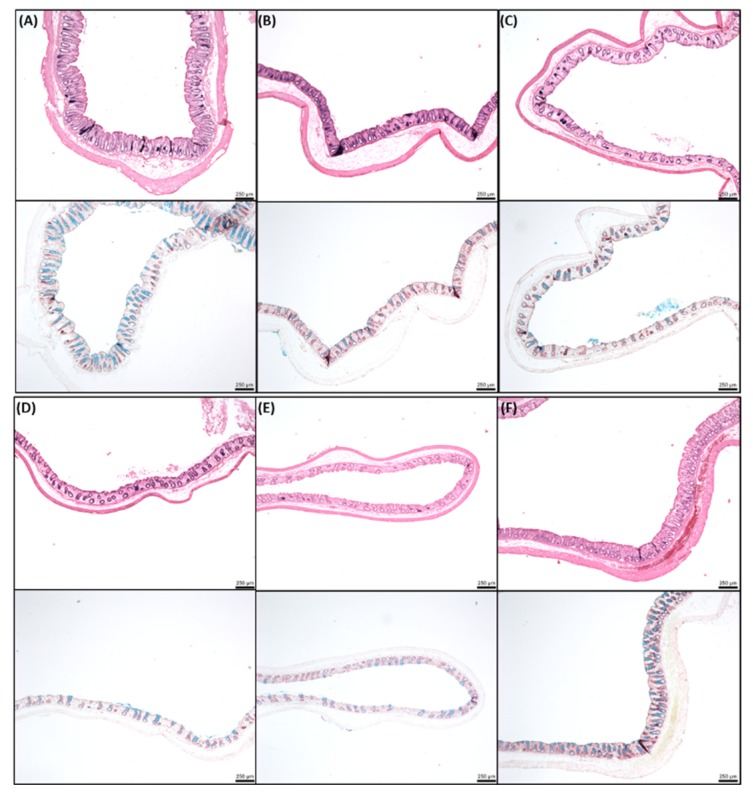
Representative histology obtained after intra-colonic instillations for 120 min. H&E (upper panels, (**A**–**F**)) and alcian blue (lower panels). (**A**) insulin (50 IU/kg); (**B**) insulin/1 mM SL; (**C**) insulin/10 mM SL; (**D**) insulin/25 mM SL; (**E**) insulin/50 mM SL; (**F**) insulin/25 mM C_10_. Bar = 250 µm.

In colonic tissue following instillations, high concentrations of SL with insulin caused some minor epithelial damage (Figure 9), but the barrier was maintained in each case. From concentrations of 25 mM SL and upwards, some cell sloughing was observed. The quality of the tissues was much higher than those obtained from Ussing chamber studies, reflecting the importance of blood supply for maintaining good viability. It was apparent from the sections that the colonic tissue from instillations was generally in better condition than jejunum instillations. Finally, additional colonic instillation controls were performed in a separate study with insulin/C_10_ (10 mM) and showed acceptable histology at 120 min (Figure S3), similar to that seen with SL (25 mM).

## 4. Discussion

Sucrose laurate is a non-ionic food-grade surfactant that meets many of the criteria for a useful oral PE. The low critical micelle concentration (CMC)and non-ionic nature of SL makes it attractive in self-emulsifying systems, as the concentration needed to reduce interfacial tension is lower than that of ionic detergents [42]. SL can also add the solubilisation of the active ingredient in such a system. The aim of this study was to systematically evaluate it as an intestinal permeation enhancer (PE), in vitro, ex vivo and in vivo. Attempts were also made to obtain a better understanding of the mechanism of action of SL. Sodium caprate (C_10_), a known PE, was used as a positive control.

Initial studies were carried out in Caco-2 monolayers in HBSS to determine a concentration range at which SL increased the *P*_app_ of [^14^C]-mannitol. The concentration range included its CMC in water of 0.36 mM [43], as medium-chain fatty acids are known to enhance permeability around this value [44]. Differences among reported CMCs for SL in the literature can relate to buffer selection, degree of esterification, incubation temperature, and analytical methods. For example, the difference in CMC values for SL in water compared to Transcutol^®^ were 60 fold (0.05% *w*/*v* compared to 3%, respectively) [45], which has implications for quantities that may be incorporated in emulsion formulations. Still, the CMC for non-ionic surfactants is relatively unaffected by the salt content of buffers. The related non-ionic surfactant sugar esters, tetradecyl maltoside (TDM) and coco-glucoside, had similar CMC values across several physiological buffers [39,46].

The sodium salt of lauric acid (C_12_) has up to a 10 fold higher CMC than SL. For C_12_, the CMC was 2.75 mM in calcium-free DMEM [47], while values of 1.58 [48] and 1.7 mM [44] were obtained in calcium-free HBSS. We hypothesised that the lower CMC for SL might confer higher potency as a PE compared to C_10_ and C_12_. Sucrose laurate at 0.5 mM transiently reduced TEER, and at 1 mM it irreversibly reduced TEER and increased *P*_app_ of [^14^C]-mannitol across Caco-2 monolayers. Reduction in TEER was seen at a concentration near its CMC. 1 mM SL appeared to be a tipping point for Caco-2 monolayers in that the TEER was not recoverable and which is backed up by the MTS assay in cells where 2.5 mM caused a reduction in the signal at 60 min exposure. A reversible reduction in TEER indirectly indicates that the tight junctions between cells are opening and that hydrophilic macromolecules can be more easily transported across the epithelium by the paracellular route.

In a study on RPMI 2650 nasal epithelial monolayers, a transient reduction in TEER was seen at 0.2 mM SL (below the CMC), and an irreversible reduction in TEER was seen at 0.6 mM at 60 min [24]. The trends observed are similar to those seen here, where the *P*_app_ of FD4 increased 14 fold at the highest concentration of SL tested in the nasal cell monolayers. The RPMI 2650 cell viability assays also detected toxicity at 0.6 mM SL, confirming a close association between membrane damage and increased flux at the same concentration. In other studies, using Caco-2 monolayers, 180 µg/mL (~0.34 mM) SL reduced TEER by ~45% [49], while Kiss et al. [25] detected a reduction in TEER by ~25% upon exposure to 100 µg/mL (0.2 mM). In the current study, no TEER reduction was seen at 0.05 mM in contrast to 0.5 mM SL. Overall, concentrations around CMC 0.35 mM seemed to be the target for non-cytotoxic permeation enhancement for SL in monolayers, as indicated by reversible TEER reduction and increases in *P*_app_ of markers, but this in vitro “therapeutic index” was very low. When C_12_ was previously examined as a PE in Caco-2 transport studies and compared to the family of medium-chain fatty acid sodium salts (MCFA) with chain lengths of C_8–11_, it proved the most potent but most cytotoxic [12]. Increases in the *P*_app_ of [^14^C]-mannitol were seen at 2 mM C_12_, whereas cytotoxicity was seen at 5 mM. In sum, although SL has a lower CMC than C_12_, both agents reduced TEER, increased *P*_app_ and induced cytotoxicity at similar concentrations. Finally, to put these in vitro data in context, lauroyl carnitine chloride (LCC) is in a formulation in clinical trials for oral peptides [3]. The CMC of LCC is ~1.2 mM which is 3.5 times that of SL [50], but an MTS assay showed toxicity at 1 mM (24 h) and at 2.5 mM (1 h) in Caco-2 cells [51]. This is similar to what was seen here for SL and it confirms the problems in attempting to discriminate permeability enhancement from cytotoxic events in cell models.

To further explore the mechanism of sub-lethal changes induced by SL on Caco-2 cells we examined a range of cellular parameters by quantitative HCA, which detects changes in live cells at an order of magnitude more sensitive than standard end-point cytotoxicity assays [52]. One of the parameters examined was MMP, as a decrease is seen as a prerequisite for apoptosis [53,54]. If cell capacity to generate ATP is reduced, it is accompanied by a reduction in MMP, which leads to the release of apoptotic factors. These include cytochrome c which induces the formation of apoptosomes to activate proteolytic caspases [53]. A reduction in MMP should also correlate with data from the MTS assay [55], and this was the case here for SL. The Caco-2 cells initially displayed an increase in the MMP from 0.05–0.1 mM SL at 1 h and 24 h, a stress-induced compensating mechanism thought to be due to the fact of hormesis [52]. At 1 mM and higher concentrations of SL, a decrease in the MMP was seen at both time points which tallied with the MTS data. As expected of a solubilizing surfactant, the PMP of cells was increased at 0.5 mM SL (24 h) and 1 mM at 1h, consistent with plasma membrane fluidisation [56] as well as the increase in *P*_app_ and irreversible reductions in TEER across monolayers seen at this concentration. Mitochondrial-mediated apoptosis and detergent damage are therefore likely to both occur when Caco-2 cells are exposed to SL.

Sucrose laurate did not cause an increase in IC in Caco-2 cells even at 10 mM for 24 h. This result is similar to that observed with non-ionic surfactant, TDM [39], whereas another, Kolliphor HS15, increased IC at 50 mM [56]. Since C_12_ increased IC at 1 mM [12], this was surprising, although there are a number of possible explanations. In theory, an increase in MMP should cause an increase in IC as calcium sequestered in mitochondria is released to the cytosol, but perhaps the mitochondria were not damaged sufficiently to trigger this event. Indeed, an increase in cytosolic calcium is considered a late event in apoptosis [57], so it possible that the cells were in the early stages. A second possible source of an increase in IC is the influx of extracellular calcium due to the damage to the plasma membrane. Cavanagh et al. [56] measured IC levels over time after exposure to 50 mM Kolliphor and noted an initial increase in IC within the first 3 min of exposure which then returned to basal levels within 20 min. Conversely, it is possible that the extent of the damage induced by SL was so high that it caused the Fluo-4 dye to leak out of the cell thereby yielding an artefact in the signal [52]. This scenario is unlikely, as the increases in PMP were similar to those seen with 8.5 mM concentrations of C_10_ in Caco-2 cells, where large increases in IC were induced [12]. HCA therefore identified differences in IC patterns following exposure to SL or medium-chain fatty acid sodium salts, suggesting that their mechanism of action is not quite the same.

According to immunofluorescence, SL disrupted the TJ protein ZO-1 in Caco-2 cells. This protein is integral to the assembly and maintenance of tight junctions, as it has physical contact with most other junction proteins [58]. Disruption in continuous ZO-1 signalling began to appear at 0.5 mM SL. Others observed no effect of SL (0.2 mM) on F-actin, ZO-1, claudin-1 and β-catenin in Caco-2 cells [25], a concentration lower than those used here. The changes induced by SL on ZO-1 in the current study are therefore consistent with an increase in paracellular flux of [^14^C]-mannitol and reduction in TEER, while the HCA data support an initial membrane perturbation mechanism for the surfactant. When Kolliphor-HS15 was tested in Caco-2 cells, it increased plasma membrane fluidity [55] that was ascribed to stress induced by heat shock, in turn linked to cytotoxic mitochondrial-mediated apoptosis. These authors argued that membrane fluidization is the initial mechanism induced by all non-ionic surfactants including alkyl maltosides and polysorbates. This analysis also fits the current study for SL, as it can account for the downstream events at tight junctions. Ultimately, while Caco-2 monolayers are ideal for initial investigations to determine whether a putative PE performs and yields mechanistic data, they give little information about potential oral formulations for humans. Caco-2 monolayers lack mucus to protect themselves from surfactant-based enhancers, and they lack an intact blood supply needed for repair [59], and these are the main reasons why cytotoxicity was seen with SL at relatively low concentrations. However, it may be that at the high concentrations used in vivo, its main mechanism is to fluidise the plasma membrane and to act as a detergent rather than to specifically enhance paracellular flux.

Isolated colonic mucosae mounted in Ussing chambers have previously been used as a model to examine PEs. A pattern is that higher concentrations of PEs are generally required to induce permeability compared to Caco-2 monolayers [39,46]. Tissue mucosae can also tolerate higher concentrations better than monolayers, but some histological damage is still invariably present even in controls mounted over 120 min [4]. However, muscle-stripped small intestinal tissue is more difficult to consistently dissect and is less robust than colonic mucosae in Ussing chambers, so isolated jejunal mucosae are too variable for screening PEs. Nevertheless, colonic epithelial transport should still have some relevance to other regions. At 1.5–10 mM concentrations, SL increased the *P*_app_ of both FD4 and [^14^C]-mannitol and reduced TEER in a concentration-dependent fashion, similar to Caco-2 monolayers but at higher concentrations. These SL concentrations did not impede the functional capacity of carbachol to induce electrogenic chloride secretion, even though there was evidence of some epithelial structural perturbation at 10 mM. A PE with an extensive [30] record in oral peptide formulations in clinical trials, C_10_, was used as a benchmark and we found that SL demonstrated similar efficacy at 10 mM, but that C_10_ induced more membrane damage. The non-ionic surfactant PE, dodecyl maltoside (DDM), is similar in structure to SL but with maltose replacing sucrose. Dodecyl maltoside (10 mM) induced an enhancement ratio (ER) for 6.0 for [^14^C]-mannitol and 16.4 for FD4 across isolated colonic tissue [39]. These are higher than the ER values obtained here for 10 mM SL—2.6 for [^14^C]-mannitol and 8.2 for FD4—although the stimulated absolute *P*_app_ values in both studies were similar. Overall, the data confirm that SL is at the upper end of PE efficacy as initially suggested in a pre-screen of a selection of PEs in rat colonic mucosae [27].

Sucrose esters including SL have been studied as a potential PE for oral delivery of impermeable small molecules using rat intestinal instillations [28,29]. Jejunum was chosen here as the initial small intestinal region to focus on. In comparison to other regions of the small intestine, it is the longest, has the longest transit time, and has the largest overall surface area [60]. When FD4 was ad-mixed with SL at set distances from the stomach pylorus, plasma levels suggested that SL should indeed perform best in the upper part of the small intestine [30]. In the current study, insulin and 100 mM SL were co-administered intra-jejunally and exhibited a relative *F* of 2.5% for insulin compared to s.c. injection. It is not a particularly high value for insulin, but, nonetheless, it was only slightly less than that obtained for an equimolar concentration of C_10_ in this jejunal instillation model. In a study carried out by Onishi et al. [30], concentrations of SL from 96 mM (2.5% *w*/*v*) to 381 mM (20% *w*/*v*) were instilled intra-duodenally to rats with FD4 and yielded high plasma levels. Yamamoto et al. [29] observed the permeation enhancing effects with SL at 0.5–1% (*w*/*v*) (~10–20 mM) on the flux marker 5(6)-carboxyfluorescein when instilled to the rat small intestine. This effect at a lower concentration than that seen in Reference [30] may be due to the fact that 5(6)-carboxyfluorescein has a lower Mw than FD4. The current study was the first to examine insulin delivery with SL in the rat jejunum and, while insulin delivery was detected, it was not quite as effective as C_10_ at the same concentration in terms of % *F*, although the % PA values were similar at ~2.5%.

In intra-colonic administrations, however, SL performed better than C_10_. For SL (25 mM) when administered with insulin, the relative % *F* was 9% compared to 6% for C_10_ (10 mM). The *T*_max_ for SL was also the same as s.c. injection of insulin, which suggests that the PK profile for insulin instilled with SL in colon is similar to s.c. injection, albeit at a 50 fold higher concentration. Instillation of insulin with higher concentrations of SL (50 mM) and C_10_ (25 mM) did not further increase the bioavailability of insulin. This suggests that once a maximum concentration is achieved, increasing the concentration of these PEs does not further increase delivery. Above the CMC, micelles formed by the surfactant-based PEs may form aggregates thereby decreasing their capacity to interact with the lipid bilayer of the intestinal epithelia [61]. The study carried out by Muranushi et al. [62] also compared C_10_ with sucrose caprate in promoting absorption of insulin and found them comparable in reducing blood glucose when rectally administered to rats. Our data with SL and C_10_ is also in line with research demonstrating that surfactant-type PEs generally work better in the colon compared to the small intestine [63,64]. Note that we are not advocating this route for systemic peptide delivery due to the long and variable transit time required for initiation, the complexity around tablet dissolution, and the interactions with the colonic microbiome. The higher efficacy in the colon is likely due to the fact that the jejunum is routinely exposed to high concentrations of bile salt emulsifiers [65], and its plasma membranes are likely to better withstand surfactant-type PEs. Other possible reasons for the difference are that the colon has a longer residence time and a lower level of peptidases than small intestinal regions [64,66].

Concerns have been raised as to whether chronic use of surfactant-based PEs cause irreversible damage to intestinal tissue that can be linked to allergy and immune conditions [4]. Examination of the gross histology in our in situ instillation study found only minor damage after administration of relatively high concentrations of SL to jejunum and colon. A comparison of intestinal tissue exposed to SL in Ussing chambers and intestinal instillations showed that less damage was apparent in the in vivo samples even at 10-fold higher concentrations. When the LDH assay was performed on small intestinal tissue after in situ closed loop instillations of SL (0.5% *w*/*v*, 10 mM), no increase in release was seen [29], although that concentration was lower than those used in jejunum. While cell sloughing also occurs in vivo, this damage is repairable, as the intestine has a high capacity for rapid restoration following exposure to a range of challenges. Approximately 10^11^ epithelial cells are shed every day from the small intestine, with the entire epithelium turned over every 3 days [67]. Thus, the intestine benefits from a reserve of stem cells that are dormant until epithelial injury occurs, at which point they are recruited and migrate up the crypt. Numerous studies confirm that the effects on epithelia of established surfactant-type PEs in clinical trials are mild and transient [68,69]. SL can therefore be expected to perform similarly in vivo in respect of transient membrane perturbation, in keeping with its current food grade status.

## 5. Conclusions

Sucrose laurate enhanced the *P*_app_ of ^14^C-mannitol across Caco-2 monolayers at a threshold concentration of 0.5 mM, slightly above its CMC. It exerted its surfactant action by affecting PMP and MMP but not by elevating IC in Caco-2 cells. It increased the flux of FD4 and ^14^C-mannitol, in isolated colonic mucosae as well as insulin in intestinal instillations. The mechanism of inducing permeability concurred with initial observations made in prior work with Caco-2 [24]. The concentrations of SL that were effective in vitro and ex vivo were lower than those required in vivo, where in turn larger effects were seen in colonic instillations compared to jejunal instillations. In vitro cell cytotoxicity assays seem to overestimate the extent of SL’s potential toxicity, whereas tissue histology following in vivo exposure only showed slight perturbation. By demonstrating SL efficacy for insulin delivery in instillation studies in rats, this means that an initial screening via a bioassay that presents PE and payload to the intestinal epithelium without confounding factors has been achieved. SL now needs to be further tested in gavage studies in rodents and large animals in appropriate presentations. For now, we conclude that it can be added to the list of agents potentially suitable for inclusion as PEs in macromolecule emulsion dosage forms. Finally, the novel aspects of the study are (i) a complete sequential analysis of SL across three bioassays in the same study, (ii) use of HCA to elucidate the cellular mechanism of SL and (ii) the first demonstration of insulin delivery with SL in a rat intestinal instillation model.

## Figures and Tables

**Table 1:** The PK and PD data following instillation of Sucrose laurate (SL) with insulin (50 IU/kg) to rat colon (i.c.) or jejunum (i.j.). Relative bioavailability (% *F*) and pharmacological availability (% PA) were calculated relative to s.c. injection of insulin (1 IU/kg). Statistical analysis is shown in Tables S2–S5.

Treatment	*C*_max_ (mU/L)	*T*_max_ (min)	*AUC*_(0–120)_ (mU/L·min)	% *F*	% PA
1 IU/kg (s.c.)	111 ± 26	20 ± 0	6936 ± 2436	-	-
50 mM SL (i.j.)	118 ± 74	20 ± 16	4616 ± 1171	1.3	1.7
100 mM SL (i.j.)	156 ± 80	30 ± 13	8782 ± 3007	2.5	2.4
50 mM C_10_ (i.j.)	276 ± 54	43 ± 12	15,210 ± 5831	4.4	2.8
100 mM C_10_ (i.j.)	215 ± 57	43 ± 13	11,348 ± 4489	3.3	2.5
1 mM SL (i.c.)	179 ± 30	73 ± 13	9382 ± 2400	2.7	1.9
10 mM SL (i.c.)	241 ± 59	37 ± 13	14,679 ± 6068	4.2	2.5
25 mM SL (i.c.)	459 ± 52	23 ± 3	30,954 ± 4087	8.9	2.7
50 mM SL (i.c.)	216 ± 2	33 ± 7	14,456 ± 2167	4.2	2.9
10 mM C_10_ (i.c.)	293 ± 30	47 ± 0	21,395 ± 6966	6.2	2.1
25 mM C_10_ (i.c.)	304 ± 52	37 ± 7	16,138 ± 5476	4.7	2.6

## Supplementary Materials

The following are available online at https://www.mdpi.com/1999-4923/11/11/565/s1, Figure S1: (A) Reduction in blood glucose after s.c. injection of insulin (1 IU/kg) or PBS. A statistically significant reduction in blood glucose was observed from 20 min onward using two-way ANOVA with Bonferroni’s *post-hoc* test; *** *p* < 0.001 versus PBS, (B)Serum insulin following s.c. injection; *p* < 0.01 versus PBS from 20 min. Mean ± SEM and *n* = 6 per group in each panel. Figure S2: (A) Blood glucose and (B) Serum insulin levels after intra-colonic (i.c.) instillation of insulin/SL and insulin/C_10_, and intra-jejunal (i.j.) instillation of insulin/C_10_. The insulin dose was 50 IU/kg. Symbols in (B) also apply to (A). These three concentrations of SL and C_10_ were tested along with those in Figure 7 and are shown separately here in order to make Figure 7 clearer. They are included in Table 1 and in the associated statistical analyses. Figure S3: Representative histology obtained after i.c. instillation of insulin/C10 (10 mM) after 120 min. H&E (left panel) and alcian blue (right panel). Bar = 250 µm. Table S1: Statistical analysis of % Initial TEER following apical addition of SL to rat colonic mucosae with respect to data in Figure 5A. Values were obtained using two-way ANOVA with Bonferroni’s *post-hoc* test. ** *p* < 0.01 and *** *p* < 0.001 compared to KH buffer; ns: not significant. Table S2: Statistical analysis of the reduction in blood glucose after i.j. instillation of insulin with SL using two-way ANOVA with Bonferroni’s *post-hoc* test. The data refer to Figure 7A. Insulin/C_10_ was the positive control. * *p* < 0.05, ** *p* < 0.01 and *** *p* < 0.001 compared to insulin solution control (50 IU/kg); ns: not significant. Table S3: Statistical analysis of serum insulin (mU/L) after i. j. instillation of SL with insulin using two-way ANOVA with Bonferroni’s post-hoc test. The data refer to Figure 7B. Insulin/C_10_ was used as a positive control. * *p* < 0.05, ** *p* < 0.01 and *** *p* < 0.001 compared to insulin solution controls (50 IU/kg); ns: not significant. Table S4: Statistical analysis of blood glucose after i.c. instillation of SL with insulin using two-way ANOVA with Bonferroni’s *post-hoc* test. The data refer to Figure 7C. C_10_ was used as a positive control. * *p* < 0.05, ** *p* < 0.01 and *** *p* < 0.001 compared to insulin solution control (50 IU/kg); ns: not significant. Table S5: Statistical analysis of serum insulin (mU/L) after i.c. instillation of SL with insulin using two-way ANOVA with Bonferroni’s *post-hoc* test. The data refer to Figure 7D. Insulin/C_10_ was used as a positive control. * *p* < 0.05, ** *p* < 0.01 and *** *p* < 0.001 compared to insulin control (50 IU/kg); ns: not significant.
