# Supplementary material for: Evaluation of Sucrose Laurate as an Intestinal Permeation Enhancer for Macromolecules: Ex Vivo and In Vivo Studies
Source: Pharmaceutics. 2019 Oct 31;11(11):565. doi: 10.3390/pharmaceutics11110565 (PMC6921008; doi:10.3390/pharmaceutics11110565)
Supplement: Supplementary file 1 [file pharmaceutics-11-00565-s001.pdf]

pharmaceutics

# Evaluation of Sucrose Laurate as an Intestinal Permeation Enhancer for Macromolecules: Ex Vivo and In Vivo Studies: Supplementary Information

**Fiona McCartney, Mónica Rosa and David J. Brayden**

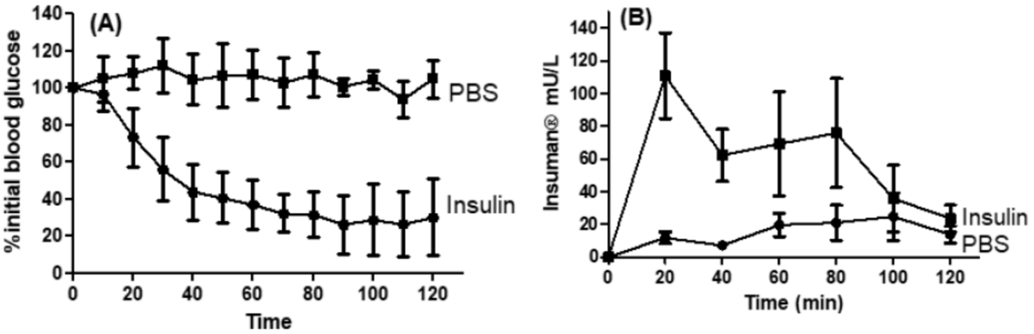


**Figure S1.** (**A**) Reduction in blood glucose after s.c. injection of insulin (1 IU/kg) or PBS. A statistically significant reduction in blood glucose was observed from 20 min onward using two-way ANOVA with Bonferroni's *post-test*; $^{***}p < 0.001$ versus PBS, (**B**) Serum insulin following s.c. injection; $p < 0.01$ versus PBS from 20 min. Mean ± SEM and $n = 6$ per group in each panel.

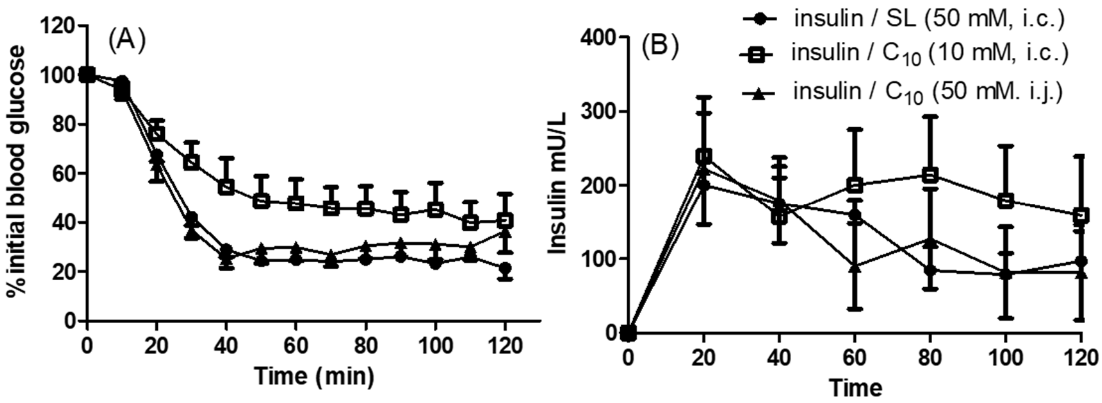


**Figure S2.** (**A**) Blood glucose, (**B**) Serum insulin levels after intra-colonic (i.c.) instillation of insulin/SL and insulin/$C_{10}$, and intra-jejunal (i.j.) instillation of insulin/$C_{10}$. The insulin dose was 50 IU/kg. Symbols in (**B**) also apply to (**A**). These three concentrations of SL and $C_{10}$ were tested along with those in Figure 7 and are shown separately here order to make Figure 7 clearer. They are included in Table 1 and in the associated statistical analyses.

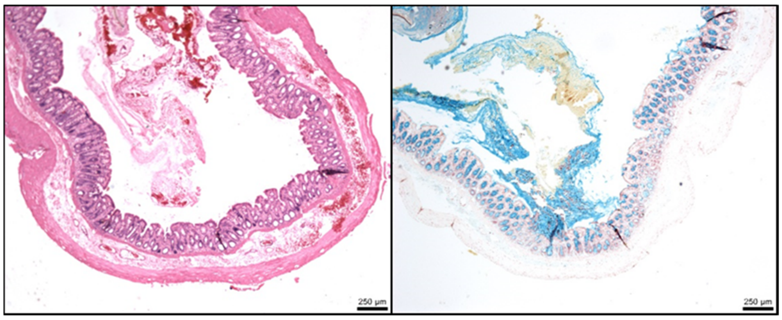

**Figure S3.** Representative histology obtained after i.c. instillation of insulin/$C_{10}$ (10 mM) after 120 min. H & E (left panel) and Alcian blue (right panel). Bar = 250 μm.

**Table S1.** Statistical analysis of % Initial TEER following apical addition of SL to rat colonic mucosae with respect to data in Figure 5A. Values were using two-way ANOVA with Bonferroni's *post-test.* **$p$ < 0.01 and ***$p$ < 0.001 compared to KH buffer; ns: not significant.

| Time (min) | 1.5 mM SL | 5 mM SL | 10 mM SL | 10 mM $C_{10}$ |
|---|---|---|---|---|
| 0 | ns | ns | ns | ns |
| 5 | *** | *** | *** | ** |
| 10 | *** | *** | *** | *** |
| 15 | *** | *** | *** | *** |
| 20 | *** | *** | *** | *** |
| 40 | *** | *** | *** | *** |
| 60 | *** | *** | *** | *** |
| 80 | *** | *** | *** | *** |
| 100 | *** | *** | *** | *** |
| 120 | *** | *** | *** | *** |

**Table S2.** Statistical analysis of the reduction in blood glucose after i. j. instillation of insulin with SL using two-way ANOVA with Bonferroni's *post-test*. Data refers to Figure 7A. Insulin/$C_{10}$ was the positive control. *$p$ < 0.05, **$p$ < 0.01 and ***$p$ < 0.001 compared to insulin solution control (50 IU/kg); ns: not significant.

| Time (min) | PBS | Insulin/SL (50 mM) | Insulin/SL (100 mM) | Insulin/$C_{10}$ (50 mM) | Insulin/$C_{10}$ (100 mM) |
|---|---|---|---|---|---|
| 0 | ns | ns | ns | ns | ns |
| 10 | ns | ns | ns | ns | ns |
| 20 | ns | ns | * | ns | * |
| 30 | ns | * | ** | *** | *** |
| 40 | ns | ** | *** | *** | *** |
| 50 | ns | ** | *** | *** | *** |
| 60 | ns | ** | *** | *** | *** |
| 70 | ns | ** | *** | *** | *** |
| 80 | ns | ns | ** | *** | *** |
| 90 | ns | * | ** | *** | *** |
| 100 | ns | ns | ** | *** | *** |
| 110 | ns | ns | * | *** | *** |
| 120 | ns | ns | *** | *** | *** |

**Table S3.** Statistical analysis of serum insulin (mU/L) after i. j. instillation of SL with insulin using two-way ANOVA with Bonferroni's *post-test*. Data refers to Figure 7B. Insulin/$C_{10}$ was used as a positive control. *$p$ < 0.05, **$p$ < 0.01 and ***$p$ < 0.001 compared to insulin solution controls (50 IU/kg); ns: not significant.

| Time (min) | PBS | Insulin/SL (50 mM) | Insulin/SL (100 mM) | Insulin/$C_{10}$ (50 mM) | Insulin/$C_{10}$ (100 mM) |
|---|---|---|---|---|---|
| 0 | ns | ns | ns | ns | ns |
| 20 | ns | *** | *** | *** | ns |
| 40 | ns | ns | *** | * | * |
| 60 | ns | ns | * | ns | ns |
| 80 | ns | ns | ns | ns | ns |
| 100 | ns | ns | ns | ns | ns |
| 120 | ns | ns | ns | ns | ns |

**Table S4.** Statistical analysis of blood glucose after i.c. instillation of SL with insulin using two-way ANOVA with Bonferroni's *post-test*. Data refers to Figure 7C. $C_{10}$ was used as a positive control. *$p$ < 0.05, **$p$ < 0.01 and ***$p$ < 0.001 compared to insulin solution control (50 IU/kg); ns: not significant.

| Time (min) | PBS | Insulin/SL (1 mM) | Insulin/SL (10 mM) | Insulin/SL (25 mM) | Insulin/SL (50 mM) | Insulin/$C_{10}$ (10 mM) | Insulin/$C_{10}$ (25 mM) |
|---|---|---|---|---|---|---|---|
| 0 | ns | ns | ns | ns | Ns | ns | ns |
| 10 | ns | ns | ns | ns | ns | ns | ns |
| 20 | ns | ns | * | ** | ns | ** | ** |
| 30 | ns | ** | *** | *** | *** | *** | *** |
| 40 | ns | *** | *** | *** | *** | *** | *** |
| 50 | ns | *** | *** | *** | *** | *** | *** |
| 60 | ns | *** | *** | *** | *** | *** | *** |
| 70 | ns | *** | *** | *** | *** | *** | *** |
| 80 | * | ** | *** | *** | *** | *** | *** |
| 90 | * | ** | *** | *** | *** | *** | *** |
| 100 | * | *** | *** | *** | *** | *** | *** |
| 110 | ns | *** | *** | *** | *** | *** | *** |
| 120 | ** | *** | *** | *** | *** | *** | *** |

**Table S5.** Statistical analysis of serum insulin (mU/L) after i.c. instillation of SL with insulin using two-way ANOVA with Bonferroni's *post-test*. Data refers to Figure 7D. Insulin/$C_{10}$ was used as a positive control. *$p$ < 0.05, **$p$ < 0.01 and ***$p$ < 0.001 compared to insulin control (50 IU/kg); ns: not significant.

| Time (min) | PBS | Insulin/SL (1 mM) | Insulin/SL (10 mM) | Insulin/SL (25 mM) | Insulin/SL (50 mM) | Insulin/$C_{10}$ (10 mM) | Insulin/$C_{10}$ (25 mM) |
|---|---|---|---|---|---|---|---|
| 0 | ns | ns | ns | ns | ns | ns | ns |
| 20 | ns | ns | ns | *** | ** | ** | ns |
| 40 | ns | ns | ns | *** | * | ns | * |
| 60 | ns | ns | ns | *** | * | ns | ns |
| 80 | ns | ns | ns | *** | ns | * | ns |
| 100 | ns | ns | ns | ns | ns | ns | ns |
| 120 | ns | ns | ns | ns | ns | ns | ns |
